# Impact of complex medication regimens on adherence in multimorbidity senior citizen – Study from Nepal

**DOI:** 10.1371/journal.pone.0345802

**Published:** 2026-04-20

**Authors:** Poonam Pant, Sudip Pandey, Santosh Khadka, Anju Joshi, Sajan Maharjan

**Affiliations:** 1 Pharmacy Program, CiST College, New Baneshwor, Kathmandu, Nepal; 2 Forest Biomaterials Program, Institute of Applied Science, Madan Bhandari University of Science and Technology, Chitlang, Bagmati Province, Nepal; 3 Public Health Program, CiST College, New Baneshwor, Kathmandu, Nepal; University of Naples Federico II: Universita degli Studi di Napoli Federico II, ITALY

## Abstract

**Background:**

Elderly individuals often experience multiple comorbidities, requiring the use of several pharmacological therapies to improve their quality of life and extend life expectancy. However, medication non-adherence is a common issue in this population, potentially leading to significant clinical complications and increased healthcare costs. This study aims to evaluate medication regimen complexity and adherence among senior citizens in Nepal. Specifically, it explores factors associated with medication complexity and adherence, and examines the relationship between the two variables in the elderly population.

**Methods:**

A cross-sectional descriptive study was conducted in selected wards of Chandragiri Municipality. Medication regimen complexity was assessed using the validated Medication Regimen Complexity Index (MRCI), while medication adherence was measured using Morisky Green Levine Adherence (MGLA) scale. Descriptive statistics (mean, standard deviation, frequency, and percentage) were used to summarize the data. Pearson’s chi-square test and correlation analysis were applied to examine the associations between MRCI levels and medication adherence. Ordinal logistic regression analysis was used to determine the impact of MRCI and other associated variables on adherence. A p-value <0.05 was considered statistically significant, and results were reported using adjusted odds ratios (AOR) with 95% confidence intervals (CI).

**Results:**

Out of 422 eligible participants, the majority of participants had a high MRCI score. A statistically significant association was found between MRCI and medication adherence (p < 0.05). Specific comorbidities, including diabetes mellitus, benign prostate hyperplasia (BPH), gastroesophageal reflux disease (GERD), thyroid disorder, chronic obstructive pulmonary disease (COPD), and coronary artery disease was significantly associated with increased MRCI scores. A weak positive correlation was observed between overall MRCI and adherence (r = 0.21), indicating a slight increase in adherence with increasing regimen complexity. Dosing frequency showed a strong correlation with MRCI (r = 0.92, p < 0.01) followed by total medication count, additional directions and dosage forms. In adjusted analysis, participants with higher MRCI level had significantly higher odds of non-adherence (AOR = 2.31, 95% CI: 1.03, 5.15). Additionally, individuals with ≥4 illnesses were more likely to be non-adherent (AOR = 3.82, 95% CI: 1.86–7.83) compared to those with two illnesses.

**Conclusion:**

Most participants exhibited high medication regimen complexity, which negatively impacted adherence levels. These findings underscore the importance for healthcare providers to implement strategies aimed at simplifying medication regimens and supporting adherence, particularly among elderly individuals with multimorbidity.

## Introduction

Ageing is a natural and continuous process. As the global population ages, the healthcare challenges associated with an expanding elderly demographic are becoming increasingly significant. In Nepal, individuals aged 60 years and above are recognized as senior citizens [1]. According to the Nepal Census 2021, 10.21% (2,977,318) of Nepal’s 29.1 million population were aged 60 years or older. With rising life expectancy and declining birth and death rates; the proportion of elderly people in the country is steadily increasing [2]. The elderly population grew from 1.5 million in 2001 to 2.9 million in 2021, with an annual growth rate of 3.5%, exceeding the overall population growth rate of 1.4% [3]. This demographic transition underscores ageing as an emerging national public health priority [4].

Older adults often manage multiple chronic conditions requiring several medications. This polypharmacy elevates the risk of drug-related problems and contributes to medication regimen complexity-a multifactorial construct encompassing the number of medications, dosing frequency, timing, dosage forms, and additional instructions [5]. Elevated regimen complexity has been associated with reduced medication adherence, hospital readmissions, adverse drug events, and diminished quality of life [6–8]. The MRCI is the most widely applied tool for quantifying regimen complexity [9]. It has been utilized across various clinical contexts, including among individuals with diabetes [10,11], cardiovascular diseases [12] and depression [8] as well as in hospital and long-term care settings [13–15].

Optimal medication therapy depends heavily on adherence to prescribed regimens. Adherence is influenced by socioeconomic status, age, health literacy, accessibility of pharmacy services, and particularly by regimen complexity. Greater complexity may also increase the likelihood of medication errors due to difficulties in following intricate dosing schedules [16,17]. The Morisky Green Levine Adherence (MGLA) scale is a validated and globally applied instrument for assessing medication adherence across various chronic conditions [18,19].

Although several studies in Nepal have examined medication regimen complexity and adherence among patients with chronic diseases, evidence specific to senior citizens in community settings is lacking. Given the growing elderly population—many with multiple comorbidities—understanding the relationship between medication regimen complexity and adherence is crucial. Therefore, this study aims to examine this association among senior citizens residing in Chandragiri Municipality, Nepal, to identify underlying challenges and inform strategies to enhance medication management and adherence within the community.

## Materials and methods

### Study design

A community-based cross-sectional study was conducted from November 1, 2023 to January 23, 2024 in Chandragiri Municipality, the largest municipality in Kathmandu District. The reporting of the study was performed based on ESPACOMP Medication Adherence Reporting Guideline (EMERGE) [20].

### Eligibility criteria

Individuals aged 60 years or older residing in Chandragiri Municipality who had been taking two or more medications for the management of multimorbidity for at least one month prior to the commencement of the study were considered eligible for inclusion. Participants were excluded if they declined to provide informed consent, were critically ill, unable to communicate, or did not understand the Nepali language.

### Sample size

The sample size was determined using the formula as follows;


$$n_{\text{0}}=\frac{Z^{\text{2}}pq}{e^{\text{2}}}$$

where, n is the desired sample size, Z is the standard normal deviation set at 1.96 corresponding to a 95% confidence interval (CI), p is the estimated prevalence (assumed as 0.5 to maximize the sample size), and e is the margin of error (0.05). Then the sample size is n= (1.96)20.5((1–0.5)/ (0.05)2 = 384. By adding 10% of non-reponse, the final sample size is 422.

### Study variables

Medication adherence was the dependent variable. Likewise, sociodemographic characteristics of the patient, such as age, gender, ethnicity, education, number of chronic diseases, number of medications, help with sorting medications and medication regimen complexity index was an independent variable.

### Sampling procedure

**Fig 1:**
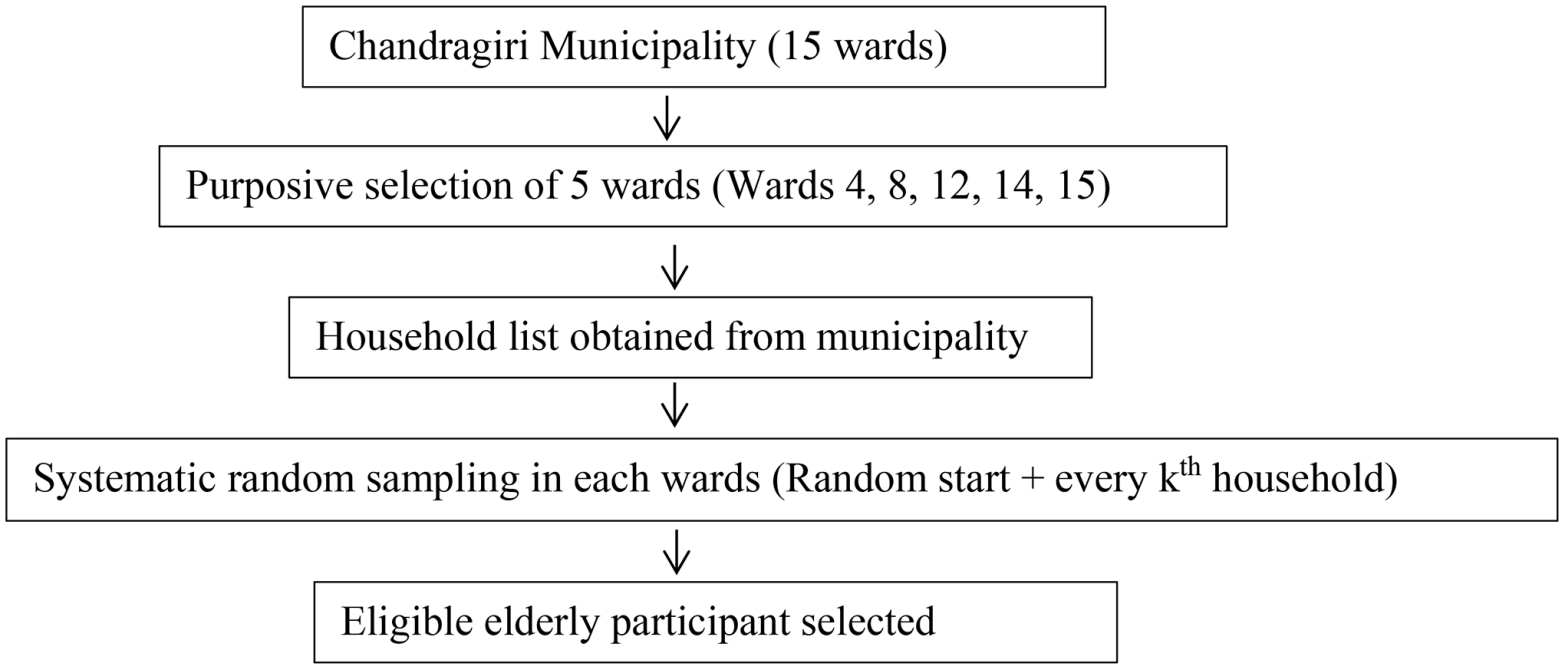
Multistage sampling procedure used to select study participants in Chandragiri Municipality.

A multistage sampling technique was used. In the first stage, five wards (Wards 4, 8, 12, 14, and 15) were purposively selected from the 15 wards of Chandragiri Municipality based on having a higher number of households, to ensure adequate representation of elderly residents. In the second stage, households within each selected ward were chosen using systematic random sampling. A complete household list was obtained from the municipal office, and an appropriate sampling interval was calculated for each ward. A random starting household was selected using a lottery method, followed by selection of every k^th^ household until the required sample size was reached. If a selected household did not have an eligible participant, the next immediate household was approached (Fig 1).

### Data collection tools and technique

A face-to-face interview was used to collect data. Information on demographics and clinical characteristics (age, gender, ethnicity, education, number of chronic diseases, number of medications and help sorting medication) were collected. The rate of medication adherence (MA) was determined using a validated instrument, the Morisky Green Levine Adherence (MGLA) score. The MGLA score is a 4-item structured instrument in which four questions have dichotomous (Yes, No) responses [21]. According to this scale, a score of 3 or 4 indicates low adherence, a score of 1 or 2 indicates a moderate level of adherence, and a score of 0 indicates a high level of adherence [22,23]. Similarly, the medication regimen complexity index (MRCI) was employed to determine the drug regimen complexity. The MRCI score is a 65-item structured instrument in which consists of three sections related to the route of drug administration (section A), dosing frequency (section B), and additional directions (section C) [9]. The sum of the scores of each of the three sections (A + B + C) contributes to a complexity index. Medication regimen complexity was divided into three categories: low, moderate, and high. With the cut-off set at ≤4 for low complexity, 5–8 for medium complexity, and a score >8 was considered as high complexity. The cut-off point was adapted from a previous study [10].

### Ethical consideration

Before data collection, permission was obtained from the office of Chandragiri Municipality and the Institutional Review Committee (IRC-CiST) at the Central Institute of Science and Technology, affiliated with Pokhara University, Nepal (IRC no.75/080/081).

### Informed consent

A written consent form was provided to the participants in Nepali and English versions explaining all the details about the study prior to data collection. For illiterate participants, the interviewer read out the content in the informed consent and obtained their thumbprint. They were informed that participation would be voluntary and that sufficient time was given to read and understand it. The identity of participants was kept confidential. No economic or other benefits were provided, and any influence was not used to collect the data from patients.

### Statistical analysis

The data were sorted, cleaned, coded, and entered into Microsoft Excel using data validation and then exported to SPSS, version 22. Descriptive statistics like frequencies, means, and percentages were used to summarize categorical and continuous variables. Pearson’s chi-square test was used to assess the association between MRCI levels and medication adherence. Correlation of MRCI with the level of medication adherence was examined using Pearson’s correlation.

### Operational definition

**Multimorbidity**: It refers to the presence of at least two chronic diseases in one individual at the same time [24].

**Medication regimen complexity (MRC)**: It represents the patient’s drug regimen, including the dosage forms, frequency, and directions for each medication provided [9].

**Medication adherence**: It is defined as patients taking their medications as prescribed. Medication adherence was assessed using the Morisky Green Levine Adherence (MGLA) score in which four questions have dichotomous (Yes, No) responses [21].

**Senior citizens**: Individuals aged 60 years and above are considered senior citizens [25].

## Results

### Social and clinical characteristics

In the study with 422 participants, the mean age of participants was (Mean ±SD: 71.94 ± 8.1) years and most of them were female 259 (61.4%). Most of the participants were aged 60–69 years 181 (42.9%) followed by aged 70–79 years 160 (37.9%), aged 80–89 years 60 (14.2%) and aged above 90 years 21 (5%). Of all participants, 195 (46.2%) were unable to read and write, 88 (20.9%) obtained informal education, 93 (22%) finished basic education, 40 (9.5%) obtained secondary education and 6 (1.4%) were undergraduate and above. Overall, 236 (55.9%) participants belonged to upper caste group, 140 (33.2%) were Janajati, 31 (7.3%) were Dalit, 15 (3.6%) were religious minorities.

In the full study sample, 235 (55.7%) individuals suffered from two illnesses and 101 (23.9%) with three illness. Most of them were diagnosed with hypertension 299 (70.9) and diabetes 199 (47.2). More than half of the individuals 247 (58.5%) consumed 2–3 medicines, 138 (32.7%) consumed 4–5 medicines and 37 (8.8%) consumed ≥6 medicines (Table 1).

**Table 1:** Socio-demographic and clinical characteristics of the participants.

Characteristics	Category	n (%)	Mean (±SD)
**Age (Years)**	60-69	181 (42.9)	71.94(±8.1)
	70-79	160 (37.9)	
	80-89	60 (14.2)	
	Above 90	21 (5)	
**Gender**	Male	163 (38.6)	
	Female	259 (61.4)	
**Education**	Unable to read and write	195 (46.2)	
	Informal education	88 (20.9)	
	Basic education	93 (22)	
	Secondary education	40 (9.5)	
	Undergraduate and above	6 (1.4)	
**Ethnicity**	Upper caste group	236 (55.9)	
	Janajati	140 (33.2)	
	Dalit	31 (7.3)	
	Religious minorities	15 (3.6)	
Comorbidity presence*	Hypertension	299 (70.9)	
	Diabetes	199 (47.2)	
	Dyslipidemia	131 (31.0)	
	BPH	79 (18.7)	
	GERD	77 (18.2)	
	Thyroid disorder	52 (12.3)	
	Musculoskeletal	51 (12.1)	
	COPD	47 (11.1)	
	Anemia	39 (9.2)	
	Asthma	32 (7.6)	
	Kidney Disease	29 (6.9)	
	Respiratory tract infection	28 (6.6)	
	Anxiety	23 (5.5)	
	Dry Eye	22 (5.2)	
**No. of Comorbidity**	≥4	59 (14)	2.53(±0.1)
	3	101 (23.9)	
	2	262 (62.1)	
**No. of medicine**	2-3	247 (58.5)	3.49(±1.6)
	4-5	138 (32.7)	
	≥6	37 (8.8)	
**Medication administration**	Self	115 (27.3)	
	Required assistance	307 (72.7)	

*Multiple response.

### Medication regimen complexity and medication adherence

The MRCI score ranged from 2 to 27, with an average score of 9.33 (±4.8). Approximately half of the population (48.1%) was categorized as high complexity, 41.5% as moderate complexity, and 10.4% as low complexity. Based on the Morisky Green Levine Adherence measuring tool, 199 (47.2%) of the respondents had low medication adherence (Table 2).

**Table 2:** Distribution of medication regimen complexity and level of medication adherence.

Characteristics	Category	n (%)	Mean (±SD)
**MRCI**	Low	44 (10.4)	9.33 (±4.8)
	Medium	175 (41.5)	
	High	203 (48.1)	
**Level of medication adherence**	High	98 (23.2)	1.96 (±1.4)
	Moderate	125 (29.6)	
	Low	199 (47.2)	

### Association between independent variables and medication adherence

Pearson’s chi-square test of association was used to identify a statistically significant association between independent variables and level of adherence, and the results revealed that patients with a higher MRCI had lower medication adherence (p **<** 0.001). Similarly, a statistically significant association was observed between age, education, medicine count, number of disease and medication administration with medication adherence whereas between age, education, medication count, number of disease with MRCI (Tables 3 and 4).

**Table 3:** Association between MRCI and medication adherence.

Medication regimen complexity	Medication adherence n (%)			*p* value
	High	Moderate	Low	
High	51 (25.1)	31 (15.3)	121 (59.6)	<0.001*
Medium	37 (21.1)	85 (48.6)	53 (30.3)	
Low	10 (22.7)	9 (20.5)	25 (56.8)	

**Table 4:** Association of independent variables with medication adherence and MRCI.

Variables	Medication adherence n (%)			*p* value	MRCI n (%)			*p* value
	High	Moderate	Low		High	Medium	Low	
**Age**								
60-69	42 (23.2)	63 (34.8)	76 (42.0)	0.010*	72 (39.8)	96 (53.0)	13 (7.2)	<0.001*
70-79	45 (28.1)	40 (25.0)	75 (46.9)		84 (52.5)	52 (32.5)	24(15.0)	
80-89	5 (8.3)	18 (30.0)	37 (61.7)		31 (51.7)	22 (36.7)	7 (11.7)	
Above 90	6 (28.6)	4 (19.0)	11 (52.4)		16 (76.2)	5 (23.8)	0 (0)	
**Gender**								0.027
Male	41 (25.2)	51 (31.3)	71 (43.6)	0.495	65 (39.9)	78 47.9)	20 (12.3)	
Female	57 (22.0)	74 (28.6)	128 (49.4)		138 (53.3)	97 (37.5)	24 (9.3)	
**Education level**								
Illiterate	45 (23.1)	52 (26.7)	98 (50.3)	0.048*	95 (48.7)	79 (40.5)	21 (12.9)	0.007*
Informal education	20 (22.7)	27 (30.7)	41 (46.6)		49 (55.7)	29 (33.0)	10 (11.4)	
Basic education	20 (21.5)	36 (38.7)	37 (39.8)		29 (31.2)	52 (55.9)	12 (12.9)	
Secondary education	10 (25.0)	7 (17.5)	23 (57.5)		27 (67.5)	12 (30.0)	1 (2.5)	
Undergraduate and above	3 (50.0)	3 (50.0)	0 (0.0)		3 (50.0)	3 (50.0)	0 (0)	
**Ethnicity**								
Upper caste	58 (24.6)	66 (28.0)	112 (47.5)	0.662	109 (46.2)	99 (41.9)	28 (11.9)	0.137
Janajati	31 (22.1)	45 (32.1)	64 (45.7)		68 (48.6)	62 (44.3)	10 (7.1)	
Dalit	4 (12.9)	11 (35.5)	16 (51.6)		15 (48.4)	10 (32.3)	6 (19.4)	
Religious minorities	5 (33.3)	3 (20.0)	7 (46.7)		11 (73.3)	4 (26.7)	0 (0)	
**No. of medicine**								
2-3	64 (25.9)	93 (37.7)	90 (36.4)	<0.001*	40 (16.2)	163 (66.0)	44 (17.8)	<0.001*
4-5	29 (21.0)	28 (20.3)	81 (58.7)		126 (91.3)	12 (8.7)	0 (0)	
>6	5 (13.5)	4 (10.8)	28 (75.7)		37 (100.0)	0 (0)	0 (0)	
**Number of Illness**								
2	72 (27.5)	85 (32.4)	105 (40.1)	<0.001*	96 (36.6)	135 (51.5)	31 (11.8)	<0.001*
3	22 (21.8)	30 (29.7)	49 (48.5)		60 (59.4)	28 (27.7)	13 (12.9)	
≥4	4 (6.8)	10 (16.9)	45 (76.3)		47 (79.7)	12 (20.3)	0 (0)	
**Medication administration**								
Self	19 (16.5)	46 (40.0)	50 (43.5)	0.010*	52 (45.2)	50 (43.5)	13 (11.3)	0.762
Required assistance	79 (25.7)	79 (25.7)	149 (48.5)		151 (49.2)	125 (40.7)	31 (10.1)	

* Chi-square (p < 0.05).

### Correlation between MRCI score and their subscores

The correlation between the total MRCI score and its subscores (dosage form, dosing frequency, and additional directions) as well as total medicine count was assessed using Pearson’s correlation coefficient. A strong positive correlation was observed between total MRCI and dosing frequency (r = 0.92, p < 0.01), total medicine count (r = 0.83, p < 0.01), and additional directions (r = 0.82, p < 0.01), while a moderate positive correlation was noted with dosage form (r = 0.56, p < 0.01). These strong correlations are expected because each subscore constitutes a component of the overall MRCI score, indicating internal consistency within the index rather than independent associations between unrelated variables (Table 5).

**Table 5:** Correlation matrix between MRCI score and their subscore.

	MRCI	Total med. Count	Dosage form MRCI	Dosing frequency MRCI	Add. directions MRCI
MRCI	1				
Total med. Count	.83^**^	1			
Dosage form MRCI	.56^**^	.15^**^	1		
Dosing frequency MRCI	.92^**^	.85^**^	.31^**^	1	
Add. directions MRCI	.82^**^	.86^**^	.11^*^	.73^**^	1

*Pearson correlation (p < 0.05).

** *Pearson correlation (p < 0.01).*

### Factors associated with medication non-adherence

Associations of MRCI and other associated variables with the level of medication adherence were examined using ordinal logistic regression, given the ordinal nature of the medication adherence levels (low, moderate, and high). The ordinal logistic regression analysis showed that participant’s number of illness and MRCI levels were significantly associated with medication non-adherence. The odds of having non-adherent with medications among individuals with ≥4 illness were increased by 3.82 times (AOR = 3.82, 95% CI: 1.86, 7.83) compared with those who had 2 illness. On the other hand, individual’s with higher MRCI level were 2.31 times more likely to be non-adherent with medications (AOR = 2.31, 95% CI: 1.03, 5.15) as compared with participants with lower MRCI level (Table 6).

**Table 6:** Association between independent variables and medication adherence using ordinal logistic regression.

Variables		COR (95% CI)	*p*-value	AOR (95% CI)	*p*-value
Age	60-69	0.84 (0.35, 2.01)	0.689	1.02(0.03, 1.59)	0.965
	70-79	0.86 (0.36, 2.10)	0.744	0.82 (0.32, 2.11)	0.682
	80-89	1.89 (0.71, 5.02)	0.202	1.92 (0.68, 5.44)	0.220
	Above 90	Ref		Ref	
Education	Illiterate	5.06 (1.17, 21.83)	0.030*	3.87(0.81, 18.44)	0.089
	Informal education	4.60 (1.04, 20.40)	0.045*	3.83(0.79, 18.56)	0.095
	Basic education	3.96 (0.90, 17.44)	0.069	3.84(0.79, 18.63)	0.095
	Secondary education	6.08 (1.27, 29.13)	0.024*	4.72(0.91, 24.56)	0.065
	Undergraduate and above	Ref		Ref	
Number of medicines	2-3	0.22 (0.10, 0.49)	0.000*	0.46 (0.16, 1.29)	0.139
	4-5	0.45 (0.19, 1.02)	0.057	0.93 (0.37, 2.32)	0.880
	>6	Ref		Ref	
Number of illness	≥4	4.83 (2.55, 9.16)	0.000*	3.82 (1.86, 7.83)	0.000*
	3	1.39 (0.90, 2.14)	0.131	0.99 (0.61, 1.57)	0.959
	2	Ref		Ref	
Medication administration	Self	1.03 (0.70, 1.53)	0.869	1.05 (0.68, 1.63)	0.816
	Required assistance	Ref		Ref	
MRCI levels	High	0.95 (0.50, 1.82)	0.883	2.31 (1.03, 5.15)	0.043*
	Medium	0.50 (0.34, 0.72)	0.000*	0.98 (0.56, 1.74)	0.955
	Low	Ref		Ref	

*statistically significant at p < 0.05; COR, crude odd ratio; AOR, adjusted odd ratio.

## Discussion

This study evaluated the relationship between medication regimen complexity and medication adherence among senior citizens with multiple comorbidities. The majority of participants were female, which is consistent with findings from similar studies conducted among patients with chronic diseases in Nepal, including those with chronic obstructive pulmonary disease (COPD) [23,24]. The higher proportion of female participants may reflect the greater life expectancy of women in Nepal, as well as their increasing engagement in healthcare-seeking behaviors. Additionally, the higher prevalence of chronic conditions such as osteoporosis and metabolic disorders among postmenopausal women could contribute to this distribution, as supported by previous studies. However, this explanation should be interpreted cautiously, as the present study did not directly investigate the biological or behavioral determinants of gender distribution.

In this study, we used a validated MRCI tool to quantify the complexity of a medication regimen among senior citizens. We have stratified MRCI scores based on low, medium, and high regimen complexity. We found that almost one-third of the population had an average of at least four to five medications prescribed per day. This finding is not surprising since nearly 90% of study participants had at least one additional ailment, such as diabetes, hypertension, dyslipidemia, benign prostatic hyperplasia, or musculoskeletal issues, which contributed to the need for additional medications. This result aligns with previous research findings that show polypharmacy related chronic conditions making it difficult for older adults to understand their medication and manage their treatment independently [26]. The majority of participants presented a high score, which indicated that they have a complex medication routine due to several factors such as the number of medications, frequency of doses, dosage forms, and additional instructions. This is the pattern found previously, in which elderly populations are more likely to have complicated medication regimens due to polypharmacy from multiple disease states [27–29]. In the current study, most participants showed strong positive correlation of MRCI score with dosing frequency and total medication count. There are several possible explanations for this correlation. Firstly, individuals who have more comorbidity may be more susceptible to a variety of symptoms that could be managed with a number of drugs. Similarly, other recent studies have also reported a strong correlation between MRCI and number of medications [30–33].

The benefit of treatment reduces as a result of poor adherence to prescribed medications. Non-adherence is thought to account for 44.1–76.5% of treatment failures, depending on the assessment method [34]. This study found that 47.2% of the study population had low adherence to their medications, with MGLA-4 score of 4. The result is comparable to other studies on hypertension [34–37], diabetes [18,38–40]. But according to some research, in some circumstances, a more difficult regimen and a more severe illness may increase patient motivation and involvement, which would ultimately result in better adherence [26]. The authors of the earlier studies suggested that older people have more comorbidity and hence perceive themselves as ill, which then drives their greater adherence [41,42].

MRC is thought to have been associated with poor medication. The present study suggested that patients with a higher level of MRC were found to have significantly lower levels of medication adherence. This finding is in line with earlier studies conducted in Italy in community-dwelling older people on chronic polypharmacy [43], India on chronic illness [44], and Georgia [45]. This could be due to several interrelated factors such as older people with cognitive impairment can interfere with remembering doses especially in regimens requiring multiple medications at different times. Managing complex medications regimen daily can feel burdensome, leading to intentional or unintentional non-adherence. Similarly, physical limitation like arthritis, tremors, or visual impairments can make tasks like opening bottles, splitting pills, or reading labels difficult. Other factors like risk of side effects, financial constraints and inadequate support system may discourage adherence.

The present study has identified the association of participants’ sociodemographic and clinical characteristics with the level of medication adherence which showed that age, education, medicine count, number of disease and medication administration were found to have a significant association with the level of medication adherence. The associated factors identified in this study were also largely consistent with results from earlier research in Pakistan [46], China [47], Thailand [48] and Portugal [49]. This may be attributed to age-related cognitive decline, poor vision, hearing impairment, physical frailty which can hinder medication-taking behavior. Additionally, forgetfulness and reduced health literacy lead to misunderstanding of medication regimens. While more comorbidity may increase polypharmacy which complicates the medication regimen, leading to confusion and unintentional omission. Similarly, the current study also assessed the association of independent variables with medication regimen complexity which revealed association of age, education, medicine count and number of disease with MRCI. This result is consistent with previous study in Norwegian population with chronic kidney disease [50]. This could be due to higher prevalence of chronic conditions and multimorbidity in older adults that necessitate multiple medications with diverse dosing schedules, routes of administration, and specific instructions (e.g., “take with food,” “avoid sunlight”). In contrast, the MRCI score was not correlated with age in the study done in South Australia [50]. This may be due to the study setting in which study was carried out that is in residential aged care facilities.

### Strengths and limitations of the study

The present study specifically targets older adults, a population particularly vulnerable to polypharmacy, regimen complexity, and poor medication adherence, making the findings highly relevant for geriatric clinical practice. The study employs standardized tools such as the Medication Regimen Complexity Index (MRCI) and validated adherence measures (e.g., Morisky Green Levine Adherence scale), enhancing the reliability and reproducibility of the results. However, the present study has several limitations. First, the MRCI was calculated solely based on information documented in medical charts. Consequently, any medications or administration instructions not recorded such as over-the-counter drugs, supplements, or patient-specific directions communicated verbally may have been missed, potentially leading to an underestimation of MRCI. Second, as the data on adherence were based partly on self-reported measures, the study is subject to recall bias and social desirability bias, whereby participants may overstate their adherence or fail to accurately recall their medication-taking behavior. Third, the study was conducted in a single region, which may limit the generalizability of the findings to other settings. Despite these limitations, the study provides valuable insights into the relationship between medication regimen complexity and adherence among older adults in the Nepalese context.

## Conclusion

This study highlights a significant association between medication regimen complexity and medication adherence among older adults. Nearly half of the elderly participants demonstrated high medication regimen complexity, and a similar proportion exhibited low adherence to their prescribed therapies. Higher MRCI scores, increased number of illnesses, and greater medication burden were all significantly associated with poorer adherence. Furthermore, the study identified strong positive correlations between MRCI and key components such as dosing frequency, total medication count, and additional instructions—factors that likely contribute to the practical challenges faced by elderly individuals in managing their regimens. Ordinal logistic regression analysis further reinforced that individuals with multiple comorbidities and those on highly complex medication regimens were significantly more likely to be non-adherent.

These findings underscore the urgent need for healthcare providers to regularly evaluate and simplify medication regimens for older adults. Interventions such as deprescribing, enhanced patient education, and the use of adherence aids may help reduce regimen complexity and improve adherence, thereby enhancing therapeutic outcomes and quality of life in this vulnerable population.

## Supporting information

S1 Data (XLSX)

S1 Checklist EMERGE Checklist. (DOCX)

## References

[1] Acharya T, Dhungana GK, Traille K, Dhakal H. Senior citizens in Nepal: policy gaps and recommendations. Gerontol Geriatr Med. 2023;9:23337214231179902. doi: 10.1177/23337214231179902 37333481 PMC10272673

[2] Government of Nepal. National Population and and Housing Census 2021. Nepal; 2021. Available from: https://censusnepal.cbs.gov.np/results

[3] National Population and Housing Census 2011. Cent Bur Stat; 2011.

[4] Chalise HN, Ghimire-Risal PK. Does population ageing affect the least developed country like Nepal? Gerontol Geriatr Med. 2018;3. Available from: https://www.researchgate.net/publication/323880154_Does_Population_Ageing_Affect_the_Least_Developed_Country_Like_Nepal

[5] Nepal Health research council. Pilot study on geriatric health issues among elderly population of Nepal. 2021.

[6] Alves-Conceição V, Rocha KSS, Silva FVN, Silva ROS, Silva DT da, Lyra-Jr DP de. Medication regimen complexity measured by MRCI: a systematic review to identify health outcomes. Ann Pharmacother. 2018;52(11):1117–34. doi: 10.1177/1060028018773691 29756471

[7] Wimmer BC, Johnell K, Fastbom J, Wiese MD, Bell JS. Factors associated with medication regimen complexity in older people: a cross-sectional population-based study. Eur J Clin Pharmacol. 2015;71(9):1099–108. doi: 10.1007/s00228-015-1883-2 26071278

[8] Linnebur SA, Vande Griend JP, Metz KR, Hosokawa PW, Hirsch JD, Libby AM. Patient-level medication regimen complexity in older adults with depression. Clin Ther. 2014;36(11):1538-1546.e1. doi: 10.1016/j.clinthera.2014.10.004 25456562

[9] George J, Phun Y-T, Bailey MJ, Kong DCM, Stewart K. Development and validation of the medication regimen complexity index. Ann Pharmacother. 2004;38(9):1369–76. doi: 10.1345/aph.1D479 15266038

[10] Ayele AA, Tegegn HG, Ayele TA, Ayalew MB. Medication regimen complexity and its impact on medication adherence and glycemic control among patients with type 2 diabetes mellitus in an Ethiopian general hospital. BMJ Open Diabetes Res Care. 2019;7(1):e000685. doi: 10.1136/bmjdrc-2019-000685 31321061 PMC6606061

[11] Rettig SM, Wood Y, Hirsch JD. Medication regimen complexity in patients with uncontrolled hypertension and/or diabetes. J Am Pharm Assoc (2003). 2013;53(4):427–31. doi: 10.1331/JAPhA.2013.13003 23892818

[12] Stange D, Kriston L, von-Wolff A, Baehr M, Dartsch DC. Reducing cardiovascular medication complexity in a German university hospital: effects of a structured pharmaceutical management intervention on adherence. J Manag Care Pharm. 2013;19(5):396–407. doi: 10.18553/jmcp.2013.19.5.396 23697477 PMC10437592

[13] Tesfaye WH, Peterson GM, Castelino RL, McKercher C, Jose MD, Wimmer BC, et al. Medication regimen complexity and hospital readmission in older adults with chronic kidney disease. Ann Pharmacother. 2019;53(1):28–34. doi: 10.1177/1060028018793419 30070583

[14] Mansur N, Weiss A, Beloosesky Y. Looking beyond polypharmacy: quantification of medication regimen complexity in the elderly. Am J Geriatr Pharmacother. 2012;10(4):223–9. doi: 10.1016/j.amjopharm.2012.06.002 22749668

[15] Advinha AM, de Oliveira-Martins S, Mateus V, Pajote SG, Lopes MJ. Medication regimen complexity in institutionalized elderly people in an aging society. Int J Clin Pharm. 2014;36(4):750–6. doi: 10.1007/s11096-014-9963-4 24906719

[16] Morisky DE, Green LW, Levine DM. Medication adherence influencing factors - an (updated) overview of systematic reviews. Med Care. 1986;24:1–17. doi: 10.1186/S13643-019-1014-82935685

[17] Morisky DE, Green LW, Levine DM. Assessing the complexity of medicine regimens-a pilot study. Med Care. 1986;24:1863–6. doi: 10.5897/AJPP11.276

[18] Pant P, Thapa S, Karkee SB, Pandey S. Prescribing pattern and medication adherence in patients with epilepsy in a Tertiary Neuro-Center, Kathmandu. Int J Epilepsy. 2024;10(01/02):028–34. doi: 10.1055/s-0044-1791263

[19] Culig J, Leppée M. From Morisky to Hill-bone; self-reports scales for measuring adherence to medication. Coll Antropol. 2014;38(1):55–62. 24851597

[20] De Geest S, Zullig LL, Dunbar-Jacob J, Helmy R, Hughes DA, Wilson IB, et al. ESPACOMP Medication Adherence Reporting Guideline (EMERGE). Ann Intern Med. 2018;169(1):30–5. doi: 10.7326/M18-0543 29946690 PMC7643841

[21] Morisky DE, Green LW, Levine DM. Concurrent and predictive validity of a self-reported measure of medication adherence. Med Care. 1986;24(1):67–74. doi: 10.1097/00005650-198601000-00007 3945130

[22] Al-Azayzih A, Kanaan RJ, Altawalbeh SM, Al-Qerem W, Smadi S. Medication adherence and its associated determinants in older adults with type 2 diabetes and cardiovascular comorbidities. Patient Prefer Adherence. 2023;17:3107–18. doi: 10.2147/PPA.S437013 38050627 PMC10693756

[23] Uchmanowicz B, Jankowska EA, Uchmanowicz I, Morisky DE. Self-reported medication adherence measured with morisky medication adherence scales and its determinants in hypertensive patients aged ≥60 years: a systematic review and meta-analysis. Front Pharmacol. 2019;10:168. doi: 10.3389/fphar.2019.00168 30930769 PMC6425867

[24] Pearson-Stuttard J, Ezzati M, Gregg EW. Multimorbidity-a defining challenge for health systems. Lancet Public Health. 2019;4(12):e599–600. doi: 10.1016/S2468-2667(19)30222-1 31812234

[25] Nepal Law Commission: Senior Citizens Act. 2021.

[26] Shareinia H, Sadeghmoghadam L, Mokhtarzadeh MR, Zahrayi SM, Jafari N, Noori R. Relationship between polypharmacy and medication adherence in the hypertensive elderly patients. Dis Diagn. 2020;9(4):153–7. doi: 10.34172/ddj.2020.05

[27] Mair A, Wilson M, Dreischulte T. Addressing the challenge of polypharmacy. Annu Rev Pharmacol Toxicol. 2020;60:661–81. doi: 10.1146/ANNUREV-PHARMTOX-010919-023508 31589822

[28] Midão L, Giardini A, Menditto E, Kardas P, Costa E. Polypharmacy prevalence among older adults based on the survey of health, ageing and retirement in Europe. Arch Gerontol Geriatr. 2018;78:213–20. doi: 10.1016/j.archger.2018.06.018 30015057

[29] Ghimire P, Pant P, Khatiwada S, Ranjit S, Malla S, Pandey S. Self-medication practice in Kathmandu Metropolitan City: a cross-sectional study. SAGE Open Med. 2023;11:20503121231158966. doi: 10.1177/20503121231158966 36896193 PMC9989370

[30] Albayrak A, Demirbaş H. Evaluation of potentially inappropriate medications use and medication complexity in elderly patients applying to community pharmacy in Turkey. BMC Geriatr. 2023;23(1):655. doi: 10.1186/s12877-023-04381-4 37833671 PMC10571236

[31] Kandel A, Pant P, Todi S, Kc S, Pandey S. Effect of exercise and pharmacotherapy on non-alcoholic fatty liver disease. SAGE Open Med. 2024;12:20503121241227090. doi: 10.1177/20503121241227090 38283643 PMC10812096

[32] Al Haqimy Mohammad Yunus MA, Akkawi ME, Fata Nahas AR. Investigating the association between medication regimen complexity, medication adherence and treatment satisfaction among Malaysian older adult patients: a cross-sectional study. BMC Geriatr. 2024;24(1):447. doi: 10.1186/s12877-024-05016-y 38778251 PMC11110348

[33] Abdu N, Idrisnur S, Said H, Kifle L, Habte N, Ghirmai S, et al. Inappropriate medication prescribing, polypharmacy, potential drug-drug interactions and medication regimen complexity in older adults attending three referral hospitals in Asmara, Eritrea: a cross-sectional study. BMC Geriatr. 2025;25(1):76. doi: 10.1186/s12877-025-05736-9 39901132 PMC11789384

[34] Foley L, Larkin J, Lombard-Vance R, Murphy AW, Hynes L, Galvin E, et al. Prevalence and predictors of medication non-adherence among people living with multimorbidity: a systematic review and meta-analysis. BMJ Open. 2021;11(9):e044987. doi: 10.1136/bmjopen-2020-044987 34475141 PMC8413882

[35] Hou Y, Zhang D, Gu J, Xue F, Sun Y, Wu Q, et al. The association between self-perceptions of aging and antihypertensive medication adherence in older Chinese adults. Aging Clin Exp Res. 2016;28(6):1113–20. doi: 10.1007/s40520-015-0516-z 26690757

[36] Mahmood S, Jalal Z, Hadi MA, Khan TM, Haque MS, Shah KU. Prevalence of non-adherence to antihypertensive medication in Asia: a systematic review and meta-analysis. Int J Clin Pharm. 2021;43(3):486–501. doi: 10.1007/s11096-021-01236-z 33515135

[37] Chang S-M, Lu I-C, Chen Y-C, Hsuan C-F, Lin Y-J, Chuang H-Y. Behavioral factors associated with medication nonadherence in patients with hypertension. Int J Environ Res Public Health. 2021;18(18):9614. doi: 10.3390/ijerph18189614 34574540 PMC8469687

[38] Sendekie AK, Netere AK, Kasahun AE, Belachew EA. Medication adherence and its impact on glycemic control in type 2 diabetes mellitus patients with comorbidity: a multicenter cross-sectional study in Northwest Ethiopia. PLoS One. 2022;17(9):e0274971. doi: 10.1371/journal.pone.0274971 36130160 PMC9491880

[39] Shimels T, Asrat Kassu R, Bogale G, Bekele M, Getnet M, Getachew A, et al. Magnitude and associated factors of poor medication adherence among diabetic and hypertensive patients visiting public health facilities in Ethiopia during the COVID-19 pandemic. PLoS One. 2021;16(4):e0249222. doi: 10.1371/journal.pone.0249222 33822807 PMC8023457

[40] Ekenberg M, Qvarnström M, Sundström A, Martinell M, Wettermark B. Socioeconomic factors associated with poor medication adherence in patients with type 2 diabetes. Eur J Clin Pharmacol. 2024;80(1):53–63. doi: 10.1007/s00228-023-03571-8 37870618 PMC10781833

[41] Lee GKY, Wang HHX, Liu KQL, Cheung Y, Morisky DE, Wong MCS. Determinants of medication adherence to antihypertensive medications among a Chinese population using Morisky Medication Adherence Scale. PLoS One. 2013;8(4):e62775. doi: 10.1371/journal.pone.0062775 23638143 PMC3636185

[42] Billups SJ, Malone DC, Carter BL. The relationship between drug therapy noncompliance and patient characteristics, health-related quality of life, and health care costs. Pharmacotherapy. 2000;20(8):941–9. doi: 10.1592/phco.20.11.941.35266 10939555

[43] Franchi C, Ardoino I, Ludergnani M, Cukay G, Merlino L, Nobili A. Medication adherence in community-dwelling older people exposed to chronic polypharmacy. J Epidemiol Community Health. 2021;75(9):854–9. doi: 10.1136/jech-2020-214238 33500324

[44] Punnapurath S, Vijayakumar P, Platty PL, Krishna S, Thomas T. A study of medication compliance in geriatric patients with chronic illness. J Family Med Prim Care. 2021;10(4):1644–8. doi: 10.4103/jfmpc.jfmpc_1302_20 34123906 PMC8144798

[45] Fernandez-Lazaro CI, Adams DP, Fernandez-Lazaro D, Garcia-González JM, Caballero-Garcia A, Miron-Canelo JA. Medication adherence and barriers among low-income, uninsured patients with multiple chronic conditions. Res Social Adm Pharm. 2019;15(6):744–53. doi: 10.1016/j.sapharm.2018.09.006 30241872

[46] Saqlain M, Riaz A, Malik MN, Khan S, Ahmed A, Kamran S, et al. Medication adherence and its association with health literacy and performance in activities of daily livings among elderly hypertensive patients in Islamabad, Pakistan. Medicina (Kaunas). 2019;55(5):163. doi: 10.3390/medicina55050163 31109105 PMC6572440

[47] Shi S, Shen Z, Duan Y, Ding S, Zhong Z. Association between medication literacy and medication adherence among patients with hypertension. Front Pharmacol. 2019;10:822. doi: 10.3389/fphar.2019.00822 31396088 PMC6664237

[48] Taibanguay N, Chaiamnuay S, Asavatanabodee P, Narongroeknawin P. Effect of patient education on medication adherence of patients with rheumatoid arthritis: a randomized controlled trial. Patient Prefer Adherence. 2019;13:119–29. doi: 10.2147/PPA.S192008 30666095 PMC6333161

[49] Gomes D, Placido AI, Mó R, Simões JL, Amaral O, Fernandes I, et al. Daily medication management and adherence in the polymedicated elderly: a cross-sectional study in Portugal. Int J Environ Res Public Health. 2019;17(1):200. doi: 10.3390/ijerph17010200 31892177 PMC6981635

[50] Parker K, Bull-Engelstad I, Aasebø W, von der Lippe N, Reier-Nilsen M, Os I, et al. Medication regimen complexity and medication adherence in elderly patients with chronic kidney disease. Hemodial Int. 2019;23(3):333–42. doi: 10.1111/hdi.12739 30779285

