# Supplementary material for: Impact of complex medication regimens on adherence in multimorbidity senior citizen – Study from Nepal
Source: PLoS One. 2026 Apr 20;21(4):e0345802. doi: 10.1371/journal.pone.0345802 (PMC13094964; doi:10.1371/journal.pone.0345802)
Supplement: S1 Checklist — (DOCX) [file pone.0345802.s002.docx]

**Impact of complex medication regimens on adherence in multimorbidity senior citizen – study from Nepal**

**ESPACOMP Medication Adherence Reporting Guideline (EMERGE)**

| **Section** | | **Item No** | | **Recommendation** | **Reported on  page No / line No** | | |
| --- | --- | --- | --- | --- | --- | --- | --- |
| **Minimum reporting criteria** | |  | |  |  | | |
|  | | 1a | | **Phases of medication adherence**: State the phase(s) of medication adherence studied (i.e. initiation, implementation, and persistence) and justify, where possible, the reasons the study focuses on this/these phase(s). | **NA** | | |
|  |  | 1b | | **Operational definition**: Provide the precise operational/working definition for each phase of medication adherence studied (i.e., initiation, implementation, and persistence). | **6** | | |
|  |  | 1c | | **Measurement**: Specify the methods of measuring medication adherence (e.g., self-report, claims data, blood sampling, electronic monitoring). Consider each phase studied (i.e., initiation, implementation, and persistence), with details on the performance of the measures (e.g., validity, reliability, and potential bias). | **5** | | |
|  |  | 1d | | **Results**: Describe the results of the analysis appropriate to each phase of medication adherence studied (i.e., initiation, implementation, and persistence). | **7** | | |
| **Abstract** | |  | |  |  | | |
|  | | 2a | | Present in the abstract, in as much detail as space permits, information on the 4 minimum reporting criteria (i.e., items 1.a- 1.d). | **1-2** | | |
| **Background/introduction** |  | |  | | |  |  |
|  | 3a | | Summarize what is known about the topic with appropriate reference to the phase(s) of medication adherence (i.e., initiation, implementation, and persistence). | | | **3** |  |
|  | 3b | | Describe the rationale and/or framework guiding the medication adherence study (e.g., theoretical framework and implementation science model). | | | **3** |  |
| **Study objectives or hypotheses** |  | |  | | |  |  |
|  | 4a | | State the study objectives or hypotheses with reference to the phase(s) of medication adherence studied and context (patient population and setting). | | | **NA** |  |
| **Methods** |  | |  | | |  |  |
| **Design & participants** | 5a | | Describe the setting in which the study was done. Refer to factors relevant to medication adherence, such as characteristics of the healthcare system, organization, and the team. | | | **3** |  |
|  | 5b | | State whether medication adherence was an eligibility criterion (e.g., inclusion/exclusion). If so, define the measures and rules used. | | | **NA** |  |
|  | 5c | | Describe routine care related to the management of medication adherence, if applicable (e.g. routine assessment of medication adherence, adherence support programs, and provider training). | | | **NA** |  |
| **Measurement** | *Please refer to item 1.c. in addition to the “Measurement” item below* | | | | |  |  |
|  | 6a | | Measurement methods can themselves affect medication adherence (e.g., questionnaires, blood sampling, and electronic monitoring). Address this problem as appropriate. | | | **11** |  |
| **Intervention (where applicable)** | 7a | | For intervention and comparator groups, describe each relevant level of the medication adherence intervention (e.g., healthcare system, organization, and provider and patient/caregiver). | | | **NA** |  |
|  | 7b | | Describe any implementation strategy that contributes to the translation (e.g., uptake, delivery, and sustainability) of the medication adherence intervention in clinical practice, if applicable. | | | **NA** |  |
| **Statistical analysis** | 8a | | If medication adherence is an outcome variable, justify the statistical methods, given the characteristics of the variable (e.g., phases of medication adherence, data type, statistical distribution, data censoring, longitudinal dependence). | | | **8** |  |
|  | 8b | | If medication adherence is an explanatory variable, describe how it is related to the outcome(s) (e.g., causal pathway, temporal sequence). | | | **NA** |  |
| **Results** |  | |  | | |  |  |
|  | *Please refer to item 1.d in addition to the “Results” items below* | | | | |  |  |
|  | 9a | | Determine whether non-participation and/or dropout are associated with non-adherence, and provide any relevant data. | | | **NA** |  |
|  | 9b | | Present sample characteristics relevant to medication adherence (e.g., those related to socio-demographics and therapy, condition, patient, caregiver, healthcare team/healthcare system). | | | **7** |  |
| **Discussion** |  | |  | | |  |  |
|  | 10a | | Discuss study strengths and limitations with reference to the phase(s) of medication adherence, where applicable (i.e., initiation, implementation, and persistence). | | | **11** |  |
|  | 10b | | Discuss the study findings in the context of existing evidence on medication adherence (e.g., theory, measurement, intervention effects). | | | **9** |  |
|  | 10c | | Discuss the generalizability (external validity) of the study findings with reference to the phase(s) of medication adherence, where applicable (i.e., initiation, implementation, and persistence). | | | **11** |  |
